# The Effects of Personality Traits on Online Rumor Sharing: The Mediating Role of Fear of COVID-19

**DOI:** 10.3390/ijerph19106157

**Published:** 2022-05-18

**Authors:** Kai Li, Jie Li, Fen Zhou

**Affiliations:** School of Journalism and Communication, Guangxi University, Nanning 530004, China; 2121391013@st.gxu.edu.cn (J.L.); 2021391064@st.gxu.edu.cn (F.Z.)

**Keywords:** online rumor sharing, the Big Five personality traits, fear of COVID-19

## Abstract

This study aims to explore the effects of personality traits on online rumor sharing during the novel coronavirus (COVID-19) pandemic and the mediating role of the fear of COVID-19 between them. We conducted this research using a web-based questionnaire distributed to 452 university students who were invited to fill it out. The partial least square structural equation modeling (PLS-SEM) method was used to test the data and model, with the yielded results demonstrating that three—extroversion, emotional instability, and conscientiousness—of the Big Five personality traits are positively related to a fear of COVID-19, with this fear positively affecting online rumor sharing. Moreover, fear of COVID-19 was found to act as a mediator between personality traits and online rumor sharing; thus, we can conclude that persons with high levels of extroversion, emotional instability, and conscientiousness are more likely to share rumors online due to a fear of COVID-19. This study furthers our understanding of the psychological mechanism by which personality traits influence online rumor sharing and provides references for anti-rumor campaigns taking place during the COVID-19 pandemic, as it identifies key groups and sheds light on the necessity of reducing people’s fear of COVID-19.

## 1. Introduction

The novel coronavirus (COVID-19) constitutes a threat to public health worldwide, and the spread of related (and often unsubstantiated) rumors also poses risks to public health [1]. Social media provide an opportunity for a large number of Internet users to participate in information sharing, with characteristics of openness, conversation, community, and connectedness [2]. In the social media era, Internet users are empowered to easily share information online, exacerbating the infodemic and causing rumors and facts to be mixed. During the COVID-19 pandemic, public health is in jeopardy through the proliferation of rumors on social media regarding the origin, transmission, mutations, treatments, long-term effects, and prevention of COVID-19, as well as the invention and side effects of vaccines. Specifically, related rumors negatively impact psychological issues in the public, weaken trust in public health institutions, and lead to inappropriate protective measures [3]. As the global COVID-19 situation continues to worsen, the spread of related rumors has been exacerbated, with the rumors becoming more violent in tone. Researchers have become concerned about this issue and studied online rumor sharing during the COVID-19 pandemic. Shen et al. [4] revealed that the features of information content (meaningfulness, interest, scariness, and personal relevance) and three motivations (fact-finding, relationship enhancement, and self-enhancement) influence individual rumor-sharing behavior online. Furthermore, Luo et al. [5] found that an individual’s online rumor-sharing behavior is significantly influenced and impacted by peer exchange, personal situations, and their overall fear of COVID-19. According to Apuke et al.’s study [6], engaging in the behavior of sharing fake news takes place under the remarkable influences of social relationships, Internet public opinion, social media information dependency, and trust in opinion leaders. Overall, previous studies have commonly concentrated on the effects of external environmental factors and environmentally-induced psychological states; therefore, there is a research gap when it comes to the potential effects of individual factors on online rumor sharing during the COVID-19 pandemic.

Personality is a relatively stable internal factor referring to individual differences in characteristic patterns of thinking, feeling, and behaving [7]. Burger [8] noted that personality is shaped by an individual’s stable behavior patterns and internal mental processes, while Markus [9] argued that individual behavior is determined by potential personality traits, free from social experience or roles. As such, the effects that potential personality traits have on individual information processing behavior have long represented a “hot spot” for researchers, with our understanding of that being further deepened through these studies [10,11]. Moreover, it must be noted that previous studies have reported that personality traits significantly relate to online rumor sharing [12]; however, seldom studies have focused on the relationship between personality traits and COVID-19-related rumor sharing. Given that the spread of rumors hinders the prevention and control of COVID-19, it is thus meaningful to study how personality traits influence (and have influenced) online rumor sharing under the COVID-19 pandemic so that we may better understand people’s online rumor-sharing behavior and resultantly develop effective rumor response strategies.

Gregory [13] stated that each personality is comprised of a diverse variety of traits and reflects consistent behavior patterns in individuals, experience characteristics, and stable intrinsic motivation. Further, scholars put forward the notion of the Big Five that covers all aspects of personality description in five dimensions, being a revolution in personality psychology [11]. As such, previous studies have found that the various factors underlying personality differ significantly and tangibly in their effects on individual cognition and the sharing of rumors; for example, the study conducted by Lai et al. [14] believed that people high in neuroticism and extroversion are more likely to believe rumors. In addition, extroversion can positively predict men’s inclination to believe in rumors, yet not that of women. This is because an individual’s belief in rumors influences their own rumor-sharing behavior [15]. As well, it must be mentioned that anxiety is a dimension of neuroticism [16], one that positively predicts online rumor sharing [17]. Fake news and misinformation are also rumors to some extent [18]. These former two points are noteworthy given that past studies have identified that personality traits significantly relate to the sharing of fake news and misinformation. According to Buchanan and Benson’s [19] study, the personality traits (e.g., agreeableness) of information receivers affect their online misinformation sharing behavior. In addition, Buchanan [20] found that online misinformation sharing is positively influenced by low agreeableness and conscientiousness, as well as high extraversion and neuroticism. Based on the above discussion, we can indirectly speculate that certain personality traits affect the inclination to share rumors online during the COVID-19 pandemic. However, considering that the outbreak of COVID-19 represents such a rare, major public health emergency, direct empirical evidence is first necessary in order to confirm which traits have specifically influenced online rumor sharing under the pandemic. Meanwhile, there is little direct evidence on whether the association between personality traits and the online sharing of rumors about COVID-19 varies with different demographics. As such, this study proposes the following questions:

Research question 1: How do personality traits influence the online sharing of rumors about COVID-19?

Research question 2: Does this relationship differ in various populations?

This research has a fourfold contribution: first, it expands the current body of research on rumors by exploring how personality traits have influenced online rumor sharing under the COVID-19 pandemic, a rare, sudden, and seemingly unpredictable public health crisis. Second, it helps deepen our understanding of the psychological mechanism by which personality traits influence online rumor sharing. Third, it enriches existing theoretical frameworks about online rumor sharing, and fourth, it provides references for anti-rumor campaigns under this and other sudden public health crises.

## 2. Theoretical Background

### 2.1. Personality Traits

Allport and Odbert [21] proposed the conception of and theories underlying personality traits in the 1930s for the first time. Nowadays, such theories regarding personality traits have been widely used in the study of human information behavior, with the notion of the Big Five personality traits being foremost (and most popular) among them [22]. The Big Five personality traits include five dimensions: emotional instability (neuroticism), agreeableness, conscientiousness, extraversion, and intellect (openness to experience) [23,24]. Table 1 shows definitions for the five personality traits. This Big Five notion was used to explore the relationship between personality traits and information searching behavior in Al-Samarraie et al.’s study [25]. Yin et al. [26] also used this theory to study the effects that personality traits have on information forwarding behavior. Moreover, this theory has also been used extensively to study people’s psychological states and coping behavior(s) throughout the COVID-19 crisis; for example, Aschwanden et al. [27] investigated people’s mental and behavioral responses during this pandemic. Carvalho et al. meanwhile [28] studied the effects personality traits have had on individual engagement with containment measures throughout this pandemic, and Nazari et al. [29] researched the relationship between personality traits and fear of COVID-19. In summary, all of these studies suggested that the Big Five personality traits are valid and reliable for measuring COVID-19-related information behavior.

### 2.2. Fear of COVID-19

Fear is an adaptive emotion that arises from the presence of perceived or identified dangers and actual threats. It is one of the most common psychological reactions for individuals faced with a sudden crisis [30]. Fear of COVID-19 is specifically induced by the extremely high infection rate and relatively high mortality rate [31]. To measure an individual’s fear of COVID-19, Ahorsu et al. [32] developed the Fear of COVID-19 Scale; Luo et al. [5] also developed a scale based on Boss et al.’s [33] study.

### 2.3. Online Rumor Sharing

Allport and Postman [34] defined a rumor as “a story or a statement whose truth value is unverified”. Rumors are seen as a threat to people’s normal lives, basic rights, and even social stability. Furthermore, Oh et al. [35] highlighted that rumors are a trigger for extreme social behavior, with the popularization of social media enabling users to browse and share all kinds of information (including rumors) anytime and anywhere, thus fostering the growth and spread of online rumors [18]. Consensus on reducing and curbing the harm caused by online rumors is growing, with many researchers having studied online rumor sharing from different perspectives [4,36,37].

### 2.4. Research Hypotheses

#### 2.4.1. Personality Traits and Fear of COVID-19

An individual’s fear of COVID-19 is related to his or her specific personality traits. In line with this, Ahmed et al. [38] identified that highly adaptive individuals experience less fear of COVID-19, While Tuman [39] posited that this fear is stronger and more highly present among healthcare workers with personalities that are prone to psychological distress and chronic stress. Nazari et al. [29] further reported that neuroticism has an effect on fear of COVID-19. Therefore, this study employs the Big Five personality traits, including extroversion, agreeableness, emotional instability, conscientiousness, and intellect, to establish the constructs of personality traits [22]. Based on that, it was hypothesized that:

**Hypothesis** **1** **(H1).** *Extroversion has a positive influence on fear of COVID-19.*

**Hypothesis** **2** **(H2).** *Agreeableness has a positive influence on fear of COVID-19*.

**Hypothesis** **3** **(H3).** *Emotional instability has a positive influence on fear of COVID-19.*

**Hypothesis** **4** **(H4).** *Conscientiousness has a positive influence on fear of COVID-19.*

**Hypothesis** **5** **(H5).** *Intellect has a positive influence on fear of COVID-19.*

#### 2.4.2. Fear of COVID-19 and Online Rumor Sharing

Fear may lead to a negative mental state and behavior [40]. For example, fear impairs a person’s ability to process complex information [32]; in a fearful state, people may not think lucidly and logically about the COVID-19-related information flooding the Internet, resulting in a situation wherein they behave irrationally based on partial or inaccurate information [33]. In fact, people share information recklessly when they are desperate for comfort and relief from their fear of COVID-19, leading online rumor sharing to ensue [41]. Luo et al. [5] also posited that fear of COVID-19 can lead to online rumor sharing and tension (e.g., anxiety, depression), which is related to the findings of Oh et al. [42], who stated that people are likely to share rumors online out of tension. Therefore, it was hypothesized that:

**Hypothesis** **6** **(H6)**. *Fear of COVID-19 has a positive influence on online rumor sharing.*

#### 2.4.3. The Mediating Role of Fear of COVID-19

According to McCrae and Costa’s [43] study, personality traits shape human behavior through related beliefs and attitudes. For example, Weiss and Deary [44] posited that people possessing a high level of neuroticism might be more inclined toward adopting health-promoting behavior due to anxiety. In fact, previous studies have found that personality traits significantly influence fear of COVID-19 [29], with this fear remarkably fueling online rumor sharing [5]. Therefore, this study considered that the fear of COVID-19 mediates the effects of personality traits on the online sharing of related rumors. Based on that, it was hypothesized that:

**Hypothesis** **7a** **(H7a)**. *Fear of COVID-19 mediates the effects of extroversion on online rumor sharing under the COVID-19 pandemic*.

**Hypothesis** **7b** **(H7b)**. *Fear of COVID-19 mediates the effects of agreeableness on online rumor sharing under the COVID-19 pandemic.*

**Hypothesis** **7c** **(H7c)**. *Fear of COVID-19 mediates the effects of emotional instability on online rumor sharing under the COVID-19 pandemic.*

**Hypothesis** **7d** **(H7d)**. *Fear of COVID-19 mediates the effects of conscientiousness on online rumor sharing under the COVID-19 pandemic*.

**Hypothesis** **7e** **(H7e)**. *Fear of COVID-19 mediates the effects of intellect on online rumor sharing under the COVID-19 pandemic*.

### 2.5. Research Model

**Figure 1:**
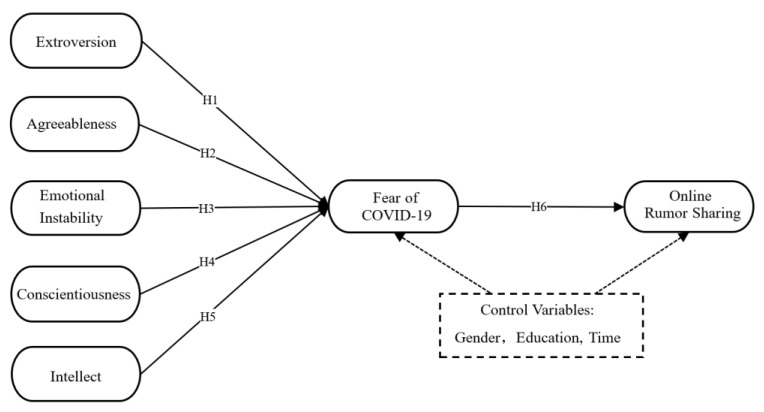
The research model. (Note: Time = Time spent on social media).

From the discussion above, we obtained the following relationships to be tested: first, personality traits influence individual fear of COVID-19; second, fear of COVID-19 influences online rumor sharing; and third, fear of COVID-19 acts as a mediator between personality traits and online rumor sharing. Control variables in this model include gender, education, and time spent on social media. Figure 1 depicts the research model of this study.

## 3. Methods

### 3.1. Measures

The three constructs—the Big Five personality traits, fear of COVID-19, and online rumor sharing—were all adopted from existing papers. Specifically, this study used a shortened version of the scale proposed by Goldberg to measure the Big Five personality traits [24], which is a common practice of using concise scales in sociological research in order to reduce respondent burden [23]. The original scale is in English, so we translated it with reference to Wang et al.’s study on the development of the Chinese Big Five personality inventory [45]. Items measuring fear of COVID-19 were taken from the scale proposed by Luo et al. [5], while items measuring online rumor sharing were taken from the scale adapted by Luo et al. [5] based on the study of Venkatesh et al. [46]. Additionally, this study used the five-level Likert scale (ranging from 1 = “not at all” to 5 = “very much”) to measure each item for the Big Five personality traits, fear of COVID-19, and online rumor sharing. Before the distribution of the questionnaire, we had requested two psychologists to review and revise the presentation of the scale. The questionnaire was piloted in a small sample of 15 individuals (university students) and improved upon based on feedback. The questionnaires filled out during the pilot were not used in the final analysis. (See Appendix A for questionnaire detail).

### 3.2. Data Collection

Wineburg et al. [47] found that university students are easily deceived by fake information on social media. Furthermore, students account for 21% of all Internet users with the highest percentage, while university students are a large part of the students in China [48]. Therefore, this study took university students as the research sample.

We created a web-based questionnaire targeting university students aged 18 or older who have self-reported having shared COVID-19-related rumors online. In order to do so, we set up a filter before answering the questionnaire so that only people who confirmed they had shared rumors could continue to fill in the following questions, and people who did not share rumors could not answer the questionnaire. The survey was carried out in China from 24 January to 23 February 2022. The data were collected by the online survey agency Wenjuanxing (www.wjx.com, accessed on 24 February 2022), which has a sample pool of more than 150 million members from diverse backgrounds. This questionnaire was randomly distributed to the participants; all of them were informed of the objectives of the study and were ensured of the anonymity of their answers and involvement prior to completing the questionnaire. A total of 660 samples were ultimately collected, and after eliminating any invalid questionnaires with incomplete or contradictory answers, 452 valid samples were prepared for data analysis. The demographic profile (gender, education, and time spent on social media) of the participants are displayed in Table 2.

This study was performed in accordance with the Declaration of Helsinki and approved by the Medical Ethics Committee of Guangxi University in response to the involvement of minimal risk and anonymous survey procedures.

## 4. Data Analysis and Results

This study analyzed the data and validated the model through the partial least square structural equation modeling (PLS-SEM) method in SmartPLS 3.2.9. The reasons for choosing the method are as follows: first, this study comprised an exploratory analysis, with PLS-SEM being more suitable for the performance of exploratory analyses compared with covariance-base structural equation modeling. Second, the PLS method is especially well-suited to the analysis of data from small samples.

### 4.1. Measurement Model

To test the measurement model, reliability, convergent validity, and discriminant validity were checked [49], with the reliability of this model being assessed using Cronbach’s α and composite reliability (CR). The Cronbach’s α values for all constructs ranged from 0.718–0.878, with the CR values ranging from 0.838 to 0.924, thus indicating that internal consistency reliability and composite reliability are satisfactory.

The Average Variance Extracted (AVE) and item loadings were used to assess convergent validity [50], with the AVE values for all constructs ranging from 0.525 to 0.803, which exceeded the threshold value of 0.5. The loadings of CONS1 (0.658) and CONS2 (0.533) used to measure conscientiousness—and the loading of EI1 (0.222) used to measure emotional instability—were found to be low, so we removed them. All of the remaining item loadings were higher than the threshold of 0.7, indicating good convergent validity. The values for reliability and convergent validity are displayed in Table 3.

The discriminant validity was assessed using the Fornell–Larcker criterion [49], according to which the square root of the AVE for each construct should be higher than the correlation of the specific construct with any of the other constructs. As displayed in Table 4, the convergent validity of the constructs in this study was good.

### 4.2. Structural Model

**Figure 2:**
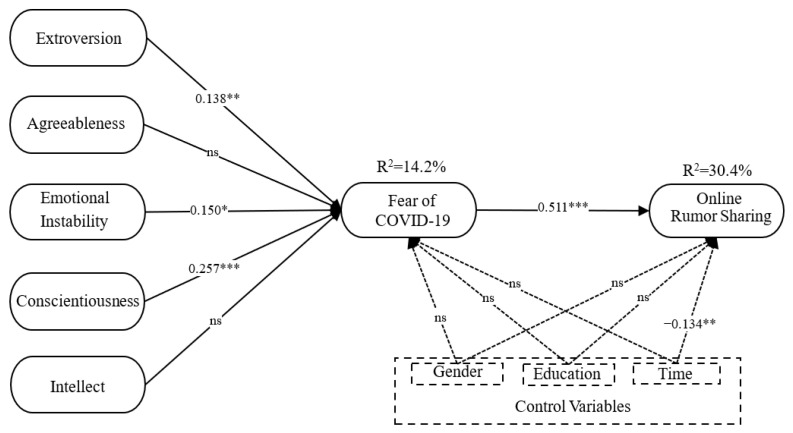
The research model. (Note: 1. *: *p* < 0.1, **: *p* < 0.01, ***: *p* < 0.001, ns: non-significant. 2. Time = Time spent on social media).

We firstly examined the path coefficient (β) and coefficient of determination (R^2^) using the PLS algorithm before then testing the significance of this model by running bootstrapping (subsamples = 5000) [49]; the results are depicted in Figure 2. For personality traits and fear of COVID-19, extroversion (β = 0.138, *p* < 0.01), emotional instability (β = 0.150, *p* < 0.1), and conscientiousness (β = 0.257, *p* < 0.001) were all found to have a positive influence on the fear of COVID-19, substantiating hypotheses H1, H3, and H4. Hypotheses H2 and H5 were not substantiated. These results indicate that university students with high extroversion, emotional instability, and conscientiousness are more likely to experience a fear of COVID-19, while university students with agreeableness and intellect feel less fear. Moreover, fear of COVID-19 (β = 0.527, *p* < 0.001) had a positive influence on online rumor sharing, substantiating hypothesis H6. It indicates that university students with a fear of COVID-19 tend to share rumors online. The control variables—gender, education, and time spent on social media—were not found to have significant relationships with the fear of COVID-19. Further, only time spent on social media was negatively related to online rumor sharing, while gender and education had non-significant relationships with online rumor sharing. Ultimately, fear of COVID-19 accounted for 14.2% of the variance in extroversion, emotional instability, and conscientiousness, while online rumor sharing accounted for 30.4% of the variance in fear of COVID-19.

This study analyzed the mediation effects according to Baron and Kenny’s study [51]. Hypotheses H7a, H7c, and H7d were substantiated, but hypotheses H7b and H7e were not. These results indicate that extroversion, emotional instability, and conscientiousness are related to online rumor sharing; meanwhile, fear of COVID-19 partially mediates between extroversion, conscientiousness, and online rumor sharing, as well as fully mediates between emotional instability and online rumor sharing; however, such fear is not a mediator between agreeableness, intellect, and online rumor sharing. See Table 5 for details.

## 5. Discussion

### 5.1. Main Findings

This study investigated the relationship of the Big Five personality traits to online rumor sharing and whether fear of COVID-19 fully or partially mediated this relationship, based on the studied sample (*n* = 452) of Chinese university students. Ultimately, this study yielded the following meaningful findings:

Hypotheses H1 and H3 were supported, indicating that people high in extroversion and emotional instability are more likely to experience a fear of COVID-19, a result that corresponds to Nazari et al.’s findings that personality traits predict this fear [29]. Hypothesis H4 was also supported, indicating that people with a high level of conscientiousness tend to also experience a fear of COVID-19, a result which differed from that of Nazari et al. [29]. Two possible reasons for this difference could be that: first, as numerous public health experts have stated, the contagiousness, danger, and severity of COVID-19 greatly exceed all expectations [52]. People with conscientiousness are highly orderly, planned, and task-oriented [53]. Nonetheless, quarantine during the COVID-19 pandemic disrupts people’s normal lives; meanwhile, due to the uncertainty of the pandemic, their scheduled tasks are impossible to accomplish in time and even have to be constantly changed, which may make them upset and fearful. Second, the two studies were conducted with different target groups. For example, this study took university students as the sample and indicated that their online rumor-sharing behavior was more likely to be affected by their personality traits. For university students, gender, education, and daily time spent on social media do not have an influence on the effects that extroversion, emotional instability, and conscientiousness have on the fear of COVID-19. Hypothesis H6 was supported, indicating that individuals suffering from a high fearfulness of COVID-19 tend to share rumors online, a result in line with the findings of Luo et al. [5]. Additionally, the relationship between fear of COVID-19 and online rumor sharing was not found to be affected by gender and education.

Hypotheses H7a and H7c were supported according to the mediation analysis, indicating that extroversion and emotional instability are related to online rumor sharing. Meanwhile, fear of COVID-19 partially mediates extroversion and online rumor sharing, as well as fully mediating emotional instability and online rumor sharing. These results are consistent with Bordia and Difonzon’s findings on people’s motivations and cognitive styles when exposed to new information [54] and also correspond to Lai et al.’s research findings on personality and rumor belief [14].

Hypothesis H7d was supported according to the mediation analysis, indicating that conscientiousness is related to online rumor sharing, and fear of COVID-19 partially mediates the relationship. This result is inconsistent with the findings of Bordia and Difonzon [54] and Lai et al. [14]; this difference could be attributed to the emergency surrounding COVID-19 and the novelty of it, as well as the numerous misreports about COVID-19 shared and broadcast by the mainstream media. For example, there was an extreme shortage of epidemic prevention items at the beginning of the COVID-19 pandemic. At that time, the Chinese mainstream media reported news about whether disposable masks could be reused after being steamed at high temperatures or sprayed with alcohol and then dried, but the conclusions in the news were self-contradictory at different stages [55]. Additionally, due to the early symptoms of COVID-19 being similar to those of the flu and pneumonia, the Chinese mainstream media misreported that Shuanghuanglian (a medicine for colds) could also be a potential treatment for COVID-19 due to a then-poor understanding of the virus. Many people ultimately believed in and widely forwarded this misinformation [56]. This could be because, in China, the mainstream media has high credibility in the public’s mind; that is to say, misinformation about COVID-19 in the mainstream media could extremely easily misguide the public and promote online rumor sharing.

### 5.2. Implications of the Study

#### 5.2.1. Theoretical Implications

First, this study extends the application areas of the Big Five personality theory by applying it to the study of rumor-sharing behavior. Previous studies on rumor sharing mainly emphasized the effects of external environmental factors [4,5,6], while few scholars focused on the internal factors of humans. This study helps in understanding the effects personality traits have on rumor sharing and the differences in rumor-sharing behavior of people with different personality traits. In other words, this study extends the application of the Big Five personality theory to a novel and significant field—online rumor sharing during the COVID-19 pandemic and reveals the process and reasons for online rumor sharing.

Second, this study sheds light on the mediating role of fear, thus contributing to our understanding of online rumor sharing. In particular, this study clarifies that personality traits affect online rumor sharing due to a fear of COVID-19 and the mediating role of fear between them. As a result, this study deepens the research on online rumor-sharing behavior by uncovering the effects that personality traits have on online rumor sharing and revealing the mechanism of COVID-19 rumor-sharing behavior on the Internet.

#### 5.2.2. Practical Implications

First, this study found that university students, especially those with high levels of extroversion, emotional instability, and conscientiousness, are more likely to share rumors online due to a fear of COVID-19. Therefore, it is suggested to identify those university students who are susceptible through personality tests so that targeted anti-rumor campaigns can be carried out preferentially.

Second, fear of COVID-19 was found to significantly affect online rumor sharing, indicating that reducing people’s fear of COVID-19 is crucial to controlling the spread of related rumors. As such, effective information communication is urgently needed to help Internet users view COVID-19 rationally so as to reduce people’s fear and curb the spread of rumors on the Internet. To be specific, the media needs to dispel rumors in time, enhance the dissemination of authoritative information, as well as convey scientific defense knowledge to the public.

### 5.3. Limitations and Future Research

As with any research, this study has been limited in some ways.

First, this study was conducted in China against the background of the COVID-19 pandemic, which may not suit the generalizability of the results. To draw more general conclusions, researchers may conduct surveys in different countries or regions under various situations in the future.

Second, the sample of this study was limited to university students and was collected through convenience sampling, which also limited the generalization of the results of our study. Therefore, future studies may try to collect data from different age and occupational groups.

Third, the shortened version of the Big Five scale was used in this study to reduce respondent burden, which is a common practice in sociological research. However, given that the concise version is limited in the number of indicators, future studies may consider using the full version to obtain more comprehensive data on personality traits.

Fourth, all of the data in this study was collected by way of self-reporting, which may involve a subjective bias. Although self-reporting has the advantages of convenience and low cost and has long been widely used in numerous studies, future studies should attempt to use different methods (e.g., psychological experiments, internet ethnography) and different types of data (e.g., objective data) to improve the results’ validity.

Fifth, we simply tested the mediating role of fear, and more work is thus needed to validate other factors which may also act as mediators, such as trust, anxiety, the third-person effect, fear of missing out, etc.

## 6. Conclusions

This study explored the relationship between personality traits, online rumor sharing, and the mediating role of fear of COVID-19. We found that individuals high in extroversion, emotional instability, and conscientiousness were more likely to experience feelings of fear related to COVID-19. Meanwhile, people experiencing this fear were inclined to share related rumors online. Furthermore, this fear was found to act as a mediator between personality traits and online rumor sharing; additionally, these relationships were not found to be influenced by gender and education. These results further our understanding of how personality traits impact the online sharing of rumors about COVID-19 and shed light on the important roles of personality traits and fear in future studies on information behavior. Practically, the results of this study can be a reference for anti-rumor campaigns.

## Figures and Tables

**Table 1:** Definitions for the Big Five personality traits [23,24].

Personality Traits	Definitions
Emotional Instability (Neuroticism)	The tendency to experience negative emotions, such as anger, anxiety, stress, and depression.
Extraversion	Pronounced engagement with the external world.
Conscientiousness	A tendency to show self-discipline and act dutifully.
Intellect (Openness to experience)	An appreciation for art, adventure, unusual ideas, and imagination.
Agreeableness	Individuals who value getting along with others

**Table 2:** Demographic profile of participants (*n* = 452).

Items	Content	Count	%
Gender	Male	186	41.2
	Female	266	58.8
Education	Undergraduate student	398	88.1
	Graduate student	54	11.9
Time spent on social media (*T* = Hour/Day)	*T* ≤ 1	5	1.1
	1 < *T* ≤ 2	39	21.5
	2 < *T* ≤ 3	97	37
	3 < *T* ≤ 4	80	17.7
	4 < *T*	231	51.1

**Table 3:** Reliability and validity analysis.

Constructs	Items	Loading	α	rho_A	CR	AVE
Rumor Sharing	RS1	0.808	0.784	0.799	0.871	0.693
	RS2	0.824				
	RS3	0.864				
Conscientiousness	CONS3	0.757	0.766	0.770	0.865	0.680
	CONS4	0.826				
	CONS5	0.808				
Emotional Instability	EI2	0.809	0.824	0.850	0.882	0.651
	EI3	0.801				
	EI4	0.785				
	EI5	0.830				
Extroversion	EXTR1	0.809	0.847	0.859	0.890	0.619
	EXTR2	0.799				
	EXTR3	0.791				
	EXTR4 EXTR5	0.813 0.717				
Intellect	INT1	0.768	0.718	0.743	0.838	0.634
	INT2	0.830				
	INT3	0.788				
Agreeableness	AGR1	0.871	0.805	0.826	0.869	0.625
	AGR2	0.741				
	AGR3	0.766				
	AGR4	0.788				
Fear of COVID-19	FEAR1	0.906	0.878	0.901	0.924	0.803
	FEAR2	0.849				
	FEAR3	0.932				

Note: α = Cronbach’s α.

**Table 4:** Assessment of discriminant validity (Fornell-Larker criterion).

Constructs	AGR	CONS	EI	EXTR	FEAR	INT	RS
AGR	**0.791**						
CONS	0.254	**0.825**					
EI	−0.131	−0.116	**0.807**				
EXTR	0.194	0.379	−0.175	**0.787**			
FEAR	0.137	0.313	0.075	0.228	**0.896**		
INT	0.403	0.478	−0.062	0.370	0.187	**0.796**	
RS	0.183	0.338	−0.099	0.335	0.527	0.291	**0.831**

Note: 1. The diagonal elements (in bold) are the square root of variance shared between the AVE, whereas the off-diagonal elements are correlations among constructs. 2. AGR = Agreeableness, CONS = Conscientiousness, EI = Emotional Instability, EXTR = Extroversion, FEAR = Fear of COVID-19, INT = Intellect, RS = Rumor Sharing.

**Table 5:** Results of the mediation analysis.

Independent Variables	Mediator	Dependent Variables	Direct Effects (T)	Indirect Effects (T)	Total Effects	VAF	Hypotheses (Result)
Extroversion	Fear of COVID-19	Rumor Sharing	0.123 ** (2.627)	0.065 ns (2.983)	0.188	34.5%	H7a supported
Agreeableness			0.031 ns (0.715)	0.027 ** (1.211)	0.059	45.7%	H7b not supported
Emotional Instability			−0.105 ** (2.642)	0.063 ** (2.848)	−0.042	−150%	H7c supported
Conscientiousness			0.044 ns (0.715)	0.119 *** (4.470)	0.181	65.7%	H7d supported
Intellect			0.124 ** (2.627)	−0.007 ns (0.291)	0.117	5.9%	H7e not supported

Note: **: *p* < 0.01, ***: *p* < 0.001, ns: non-significant.

## Data Availability

All scales used for the study are available from the corresponding author upon reasonable request. The data are not publicly available due to privacy restrictions.

## Appendix A. Scale of the Study

**Constructs**		**Measurement Items**
Extroversion	EXTR1	I see myself as someone who is the life of the party.
	EXTR2	I see myself as someone who feels comfortable around people.
	EXTR3	I see myself as someone who starts conversations.
	EXTR4	I see myself as someone who talks to many different people at parties.
	EXTR5	I see myself as someone who don’t mind being the center of attention.
Agreeableness	AGR1	I see myself as someone who sympathizes with others’ feelings.
	AGR2	I see myself as someone who has a soft heart.
	AGR3	I see myself as someone who takes time out for others.
	AGR4	I see myself as someone who feels others’ emotions.
Emotional Instability	EI1	I see myself as someone who gets stressed out easily.
	EI2	I see myself as someone who is easily disturbed.
	EI3	I see myself as someone who gets upset easily.
	EI4	I see myself as someone who changes mood a lot.
	EI5	I see myself as someone who gets irritated easily.
Conscientiousness	CONS1	I see myself as someone who is always prepared.
	CONS2	I see myself as someone who pays attention to details.
	CONS3	I see myself as someone who gets chores done right away.
	CONS4	I see myself as someone who likes order.
	CONS5	I see myself as someone who follows a schedule.
Intellect	INT1	I see myself as someone who is quick to understand things.
	INT2	I see myself as someone who spends time reflecting on things.
	INT3	I see myself as someone who is full of ideas.
Fear of COVID-19	FEAR1	I was worried about the prospect of virus infection from others.
	FEAR2	I was frightened about the prospect of virus infection from others.
	FEAR3	I was anxious about the prospect of virus infection from others.
Rumor Sharing	RS1	I have shared some virus-related rumors on social media when I did not know they were rumors.
	RS2	I have shared some virus-related rumors to my family and friends when I did not know they were rumors.
	RS3	I have shared some virus-related rumors unconsciously when I did not know they were rumors.
